# Supporting tuberculosis program in active contact tracing: a case study from Pakistan

**DOI:** 10.1186/s40249-022-00965-1

**Published:** 2022-04-09

**Authors:** Babar Tasneem Shaikh, Ahmed Khan Laghari, Sulaiman Durrani, Alina Chaudhry, Nabeela Ali

**Affiliations:** Integrated Health System Strengthening and Service Delivery (IHSS-SD) Activity, JSI Research & Training Institute Inc., Mezzanine Floor, The Grand Hotel Street 1, Sector E-11/1, Islamabad, 44000 Pakistan

**Keywords:** Active contact tracing, Community awareness, Tuberculosis, Pakistan

## Abstract

Tuberculosis (TB) is on the rise in Pakistan and there could be multiple reasons including poverty, difficulty in access to TB treatment services, non-compliance with treatment, social stigma etc. According to the TB program managers, limited treatment and testing sites for tuberculosis and lack of trained human resources play a major role in compromising TB management. A major lacuna in the TB control program is the absence of active contact tracing strategy. This is essential for a disease where positive cases are known to be able to infect a further 10‒15 individuals in a year. Tackling tuberculosis in Pakistan has been beleaguered by funding challenges and other systems’ bottlenecks such as lack of skilled human resources and insufficient supply of medicines, despite the fact that disease burden is one of the highest in the world. Although it is a notifiable disease, active case finding, contact tracing and reporting is notoriously low throughout the country. Access to diagnostics and treatment facilities has been limited and stigma attached to the disease remains deeply entrenched among the communities. Researchers have shown that enhanced and active approaches to contact investigation effectively identifies additional patients with TB among household contacts at a relatively modest cost. USAID’s Integrated Health Systems Strengthening and Service Delivery Activity extended support to the Health Departments of Sindh and Khyber Pakhtunkhwa provinces. In collaboration with the two provincial TB programs, community based active contact tracing was conducted on 17,696 individuals, based on the index cases. Among the contacts traced, 243 cases were diagnosed as drug sensitive or drug resistant TB. Awareness sessions were conducted to sensitize people on the various aspects of disease and importance of getting tested. The project also supported establishing three satellite Programmatic Management of Drug Resistant Tuberculosis (PMDT) sites for drug resistant TB treatment, enhancing the programs’ diagnostic and testing capacity.

## Background

Pulmonary tuberculosis is usually diagnosed when symptomatic individuals seek care at healthcare facilities. Historically, the outreach healthcare workers have had a minimal role in promoting the health-seeking behavior with regard to this stigmatized disease. However, some policy specialists believe the healthcare system could be more active in tuberculosis (TB) diagnosis to increase tuberculosis case detection [1]. Contact investigation, the systematic evaluation of individuals in close contact with an infectious patient, is a key active case-finding strategy for global TB control. To curb the TB epidemic, both behavior change, as well as earlier identification and treatment of infectious individuals and their close contacts, is imperative[2, 3]. TB contact investigation, defined as the systematic evaluation of people exposed (contacts) to persons who have potentially infectious TB (index cases), is a strategy to identify additional new cases of active and latent TB infection, eligible for preventive therapy. Contact investigation is initiated when a new case of TB is identified. An index case interview is performed to obtain a list of all household and non-household contacts. While procedures may vary, household visits for symptom screening are recommended for all contacts who have had cough for more than two weeks, and those who screen positive are referred for clinical evaluation for active TB, while those who screen negative are eligible to begin preventive therapy[4]. Household contacts of multidrug-resistant tuberculosis (MDR-TB) patients are at a high risk of getting infected with TB/MDR-TB, highlighting the importance of symptomatic or vulnerable individuals to be screened and treated early [5]. The contacts of diagnosed TB patients may have high prevalence of TB and can be feasibly detected through active contact tracing, but more sensitive tests than sputum smear are required [6].

Addressing disease burden of TB in Pakistan has been a challenge because of the limited funding and other systems’ bottlenecks such as dearth of skilled human resources and insufficient medicines’ availability. In spite of being a notifiable disease, its reporting is disreputably low. Limited access to diagnostics and treatment facilities and the stigma attached to the disease remains deeply entrenched among the communities [7]. In the south and south east Asian context, active contact tracing of TB cases has been employed vigorously in most countries with few exceptions [8, 9]. Pakistan stands out in this regard due to weak community engagement and action. Nevertheless, small scale research has shown that enhanced and active approaches to contact investigation effectively identifies additional patients with TB among household contacts at a relatively modest cost. These active strategies can be added to the passive contact investigation in a high burden setting to find the people with TB who are missed and meet the End TB strategy goals [10, 11].

TB is managed by the provincial TB programs (PTP) in each province of Pakistan. Despite the efforts of Sindh PTP Sindh and the support of Global Fund, PTP detected 74,524 TB cases (59%) in year 2018, out of 126,898 TB cases in the province. More significantly, 41% were hidden/unnotified cases of TB in Sindh. Currently, only 23% of index cases are receiving contact screening when this should be 100% per program standards. Similarly, in Khyber Pakhtunkhwa, case detection rate fell from 57% in 2016 to 40% in 2020 [12]. While the country’s case detection rate remains 48%, the aim of the TB program is to increase the case detection rate to 80% by 2023 [13]. It is estimated that a non-diagnosed patient with active TB, can transmit TB to at least 10 people in one year. Given the status of poverty, malnutrition, and average size of households in Pakistan, the risk of transmitting TB to other individuals increases many times, especially in rural areas. There is a dire need to address this gap through increased screening in hot spots in vulnerable rural districts, especially screening of household and other close social contacts of index cases. More accurate and credible data from rural areas might provide glimpses of actual prevalence and current burden of disease in the country. Besides estimation of the actual burden of disease, it is imperative to raise awareness about symptoms, its mode of transmission, prevention, diagnosis and treatment, and most importantly, destigmatization of the disease through health education at the grass root level [14, 15].

The Integrated Health System Strengthening and Service Delivery (IHSS-SD) Activity is a United States Agency for International Development (USAID) funded initiative in Pakistan since 2018, working closely with the federal and provincial health authorities to reorient, reform and strengthen the health system. The overall aim of this technical support was to support the Provincial TB Control Programs in Sindh and Khyber Pakhtunkhwa in improving the active contact tracing of bacteriologic positive TB cases and decentralizing the management of drug resistant TB cases. The ultimate objectives of this activity were to gather enough evidence for proving the effectiveness of active contact tracing and screening as an important strategy in improving the detection of bacteriologic positive cases of TB within the communities, and to enhance the testing capacity of the provincial TB programs. The support to TB program was rolled out on January1, 2021 and was concluded on September 30, 2021.

## Intervention to support the provincial TB program

In order to improve the case detection rate of TB in the communities, IHSS-SD Activity extended support to the TB Control Programme Sindh and Khyber Pakhtunkhwa by identifying index cases of drug resistant tuberculosis (DR-TB) and drug sensitive tuberculosis (DS-TB), tracing their contacts and screening for tuberculosis. The districts selected were Swat, Charsadda and Lakki Marwat in Khyber Pakhtunkhwa, and Qambar Shahdadkot and Larkana in Sindh. This selection was done by the provincial governments based on the disease burden in the districts.

Under usual circumstances these new cases of TB would remain undetected within their communities for prolonged periods of time and each patient of active TB could transmit the disease to approximately 10‒15 contacts in one year [3]. This activity provides evidence that tracing and screening contacts of both DR and DS TB cases can help in the early detection of unknown or undetected TB cases and reduce the transmission of TB in communities.

### Enhancing case detection rate in contacts of index DR and DS cases

Based on situation analysis and meetings with the TB control program, technical support was extended to Sindh & Khyber Pakhtunkhwa TB Control Programs to enhance the case detection rate (CDR) by tracing the close contacts of drug resistant and drug sensitive cases in the selected district. The list of index cases of DS and DR TB registered cases at PMDT site and GeneXpert sites were collected. The project field team developed a weekly plan in coordination with the District TB focal person and lady health workers under the auspices of the District Health Office. During the field visit, sputum of index cases and close contacts (only those having symptoms) were collected and submitted to the nearby PMDT site. Patients diagnosed positive were registered in the nearby PMDT/ GeneXpert site. Details of cases traced and tested, and community awareness session are provided in Table 1. In-home diagnostic services worked to limit stigma and optimize confidentiality, which was especially important for young females. The intervention also provided savings in travel time and transport money for suspected contacts.

**Table 1:** Cumulative figures from January 2021 to September 2021, capturing the IHSS-SD Activity’s support to PTP-KP & Sindh

Description	Khyber Pakhtunkhwa	Sindh
DRTB index cases visited	188	173
DSTB index cases visited	1609	682
Total index cases visited	1797	855
DRTB contacts traced	1223	1645
DSTB contacts traced	9105	5723
Total contacts traced	10,328	7368
Samples collected from DRTB contacts	752	961
Samples collected from DSTB contacts	3062	2887
Total samples collected	3814	3848
Samples found positive for DRTB	15	17
Samples found positive for DSTB	138	87
Total samples found positive	153	104
Registered positive DRTB	15	14
Registered positive DSTB	138	76
Total registered positive	153	90
Health Awareness Sessions conducted	1778	830
Participants in Health Awareness Sessions	11,837	5832

*TB* Tuberculosis,* IHSS-SD* Integrated Health System Strengthening and Service Delivery,* DR* Drug resistant,* PTP* Provincial TB Program

In total, 243 new cases were detected with positive bacteriology, comprising 29 DR and 214 DS TB cases. All of the 243 new detected cases were enrolled in their respective basic management units and programmatic drug resistant TB (PMDT) sites for management.

Some cases found positive in Sindh, refused to register at the treatment site because of lack of family support and issues of stigma due to upcoming marriage. There was also one death in Sindh, following a positive diagnosis.

### Community awareness activities

In order to augment the contact tracing and screening activities, the project field teams conducted awareness sessions on TB for the family members of the index cases and members of the community on disease prevention, symptoms, diagnosis and treatment. Table 1 above shows the total number of sessions conducted and the number of people who participated in these awareness raising sessions.

### Support for establishing satellite Programmatic Management of Drug-Resistant TB (PMDT) site

Routine management of DS TB patients is done at Basic Management Units (BMU) in the district hospitals and management of DR cases is done at the PMDT sites, also housed in the district or teaching hospitals. The satellite PMDT sites are linked with their respective large PMDT site. Hence, IHSS-SD activity supported the Health Departments of Sindh and Khyber Pakhtunkhwa provincial TB Programs to establish the satellite Programmatic Management of Drug Resistance TB Cases (PMDT) sites. The concept of satellite sites is to increase the access of presumptive TB patients to get tested in the nearby hospitals instead of going all the way to the teaching or tertiary care hospital where the main PMDT sites are operational. Therefore, one satellite site was established at the District Headquarters Hospital Qambar Shahdadkot in Sindh, and two in Khyber Pakhtunkhwa in districts Charsadda and Lakki Marwat. All the necessary equipment including GeneXpert machine was provided with the support of USAID, while the human resources were provided by the Health Departments to make these satellite sites operational.

These sites have expanded the network of PMDT sites, making it convenient for drug resistant TB patients to access lifesaving health services. These satellite sites will be attached to their respective regional PMDT sites. These sites will greatly reduce the travel and other expenditures of patients suffering from drug resistant TB. They are also expected to improve compliance to treatment and the treatment success rate that has been noted in other parts of the world. With the provision of GeneXpert machines, the testing capacity of the PMDT sites has been increased significantly.

## Discussion

TB spreads among hard-to-reach populations, and the barriers to access diagnosis and receive treatment, in addition to insufficient case identification and reporting, are slowing down the pace of World Health Organization’s End TB strategy as well as the progress towards theUnited Nations’ Sustainable Development Goals (SDGs) [4, 16]. Active case-finding or systematic screening for tuberculosis has been a vital component of the TB control programs throughout the world, used to reach out to TB patients [17]. When properly implemented, the intervention is cost effective, and helps to reduce the delays in diagnosis and treatment, and prevents further spread of disease. Patients with active TB can infect 10‒15 other people through close contact over the course of a year. When people with TB cough, sneeze, or spit, they propel TB bacteria into the air. A person needs to inhale only a few of these bacteria to become infected [18]. Moreover, the social, mental and economic burden of undiagnosed cases is catastrophic for the families [19]. There is enough evidence available that active contact tracing can limit the transmission of tuberculosis and has been much more successful than any other TB control intervention [20, 21].

For the sake of advocacy and sharing the project achievements, IHSS-SD teams have been participating in intra-district, inter-district and intra-provincial meetings on a quarterly basis, and have shared the progress of project-supported interventions with the provincial TB program managers regularly. All data related to new TB cases identified have been shared with the TB focal person based in the district health office. PTP Sindh & Khyber Pakhtunkhwa have incorporated the same activities in the next Global Fund round in order to replicate the IHSS-SD interventions.

This intervention value-added to local TB management in many ways. At present no strategy exists within the provincial TB programs regarding active contact tracing at the community level; this was only done in the IHSS-SD intervention. Based on the results of this intervention, both PTPs have decided to include the similar strategies in their upcoming round of Global fund. For the first time, household, workplace and social contacts of index TB cases were provided diagnostic services, and were linked in for registration and treatment at the TB basic management unit or the PMDT site. During the intervention, 29 DR TB cases were diagnosed. If these had remained undiagnosed and untreated, they may have transmitted disease to another 290 DR more cases (10‒15 cases/year). Similarly, 214 DS TB cases were diagnosed which may otherwise have transmitted to a further 2,140 DS cases. Moreover, community awareness sessions played a great role in educating locals on identifying symptomatic cases, and convincing suspected cases to get tested. The cost of treating a DR TB case is ten times higher than the treatment expense incurred on a DS case [22]. Intervention also supported the establishment of the PMDT site at DHQ Qambar Shahdadkot, Charsadda and Lakki Marwat through provision of necessary furniture and equipment including five GeneXpert machines, has significantly enhanced the testing capacity of the provincial TB programs.

This is a novel case study in the Pakistani context as it documents the importance of missing links in the TB program in the country. Nevertheless, this intervention had the limitations of time and financial resources, otherwise it would have been scaled up to other high disease burden districts of the two provinces.

## Conclusions

Screening of the household and other close social contacts of DS and DR-TB index cases may be given a serious consideration as a permanent feature of the TB control program in Pakistan with adequate financing and other resource inputs. This intervention has not only prevented conversion of DS TB cases into DR TB cases, but also saved the cost of treating the DR TB cases which is much higher than treating a case of DS TB. In IHSS-SD Activity’s intervention, the number of presumptive TB contacts required to be screened to identify a new DR-TB case indicate an effective strategy that could easily be scaled-up. The screening and management of vulnerable adults and even children living with TB patients should be a priority in the collective efforts to end TB in Pakistan.

## Data Availability

The data collected during the project intervention are not publicly available due to ethical and confidentiality reasons but are available from the corresponding author on reasonable request under the Ethics Committee’s approval.

## Abbreviations

CDR: Case detection rate
DR: Drug resistant
DS: Drug sensitive
IHSS-SD: Integrated Health System Strengthening and Service Delivery
MDR: Multidrug-resistant
PMDT: Programmatic Management of Drug resistant tuberculosis
PTP: Provincial TB Program
SDGs: Sustainable Development Goals
TB: Tuberculosis
USAID: United States Agency for International Development
